# A Remote Health Coaching, Text-Based Walking Program in Ethnic Minority Primary Care Patients With Overweight and Obesity: Feasibility and Acceptability Pilot Study

**DOI:** 10.2196/31989

**Published:** 2022-01-19

**Authors:** Mary H Smart, Nadia A Nabulsi, Ben S Gerber, Itika Gupta, Barbara Di Eugenio, Brian Ziebart, Lisa K Sharp

**Affiliations:** 1 Department of Pharmacy Systems, Outcomes and Policy College of Pharmacy University of Illinois Chicago Chicago, IL United States; 2 Department of Population and Quantitative Health Sciences University of Massachusetts Medical School Worcester, MA United States; 3 Department of Computer Science College of Engineering University of Illinois Chicago Chicago, IL United States

**Keywords:** mHealth, Fitbit, SMART goals, texting, health coach, mobile phone

## Abstract

**Background:**

Over half of US adults have at least one chronic disease, including obesity. Although physical activity is an important component of chronic disease self-management, few reach the recommended physical activity goals. Individuals who identify as racial and ethnic minorities are disproportionally affected by chronic diseases and physical inactivity. Interventions using consumer-based wearable devices have shown promise for increasing physical activity among patients with chronic diseases; however, populations with the most to gain, such as minorities, have been poorly represented to date.

**Objective:**

This study aims to assess the feasibility, acceptability, and preliminary outcomes of an 8-week text-based coaching and Fitbit program aimed at increasing the number of steps in a predominantly overweight ethnic minority population.

**Methods:**

Overweight patients (BMI >25 kg/m^2^) were recruited from an internal medicine clinic located in an inner-city academic medical center. Fitbit devices were provided. Using 2-way SMS text messaging, health coaches (HCs) guided patients to establish weekly step goals that were specific, measurable, attainable, realistic, and time-bound. SMS text messaging and Fitbit activities were managed using a custom-designed app. Program feasibility was assessed via the recruitment rate, retention rate (the proportion of eligible participants completing the 8-week program), and patient engagement (based on the number of weekly text message goals set with the HC across the 8-week period). Acceptability was assessed using a qualitative, summative evaluation. Exploratory statistical analysis included evaluating the average weekly steps in week 1 compared with week 8 using a paired *t* test (2-tailed) and modeling daily steps over time using a linear mixed model.

**Results:**

Of the 33 patients initially screened; 30 (91%) patients were enrolled in the study. At baseline, the average BMI was 39.3 (SD 9.3) kg/m^2^, with 70% (23/33) of participants presenting as obese. A total of 30% (9/30) of participants self-rated their health as either *fair* or *poor*, and 73% (22/30) of participants set up ≥6 weekly goals across the 8-week program. In total, 93% (28/30) of participants completed a qualitative summative evaluation, and 10 themes emerged from the evaluation: patient motivation, convenient SMS text messaging experience, social support, supportive accountability, technology support, self-determined goals, achievable goals, feedback from Fitbit, challenges, and habit formation. There was no significant group change in the average weekly steps for week 1 compared with week 8 (mean difference 7.26, SD 6209.3; *P*=.99). However, 17% (5/30) of participants showed a significant increase in their daily steps.

**Conclusions:**

Overall, the results demonstrate the feasibility and acceptability of a remotely delivered walking study that included an HC; SMS text messaging; a wearable device (Fitbit); and specific, measurable, attainable, realistic, and time-bound goals within an ethnic minority patient population. Results support further development and testing in larger samples to explore efficacy.

## Introduction

### Background

Approximately 52% of the adults in the United States live with at least one chronic disease, and 27% have multiple chronic diseases [1]. These estimates exclude obesity, which affects approximately 42% of the adults in the United States [2]. Physical activity is an important component of self-management for the most prevalent chronic diseases in the United States, such as hypertension, diabetes, asthma, and obesity [3,4]. Despite the benefits of physical activity in managing and preventing chronic disease, the rates of physical inactivity are considerable. Within the United States, rates vary by racial and ethnic groups, with 32% of Hispanic adults being physically inactive, followed by 30% of non-Hispanic Black adults and 23% of non-Hispanic White adults [5]. Even fewer reach the recommended levels of at least 150 minutes per week of moderate to intense aerobic activity (eg, brisk walking) [3]. A growing body of literature suggests that physical activity has a dose effect on health, with those who are the least active experiencing the greatest benefits [6,7]. Therefore, physically inactive individuals may experience significant health benefits by increasing the number of steps they walk [8,9].

Interventions designed to increase physical activity among people with chronic diseases in primary care have historically shown mixed results [10]. However, in 2010, the emergence of consumer-based wearables that track physical activity presented new approaches to address this problem [11,12]. Consumer-based wearables are devices that provide immediate, real-time, quantitative feedback on steps in addition to a variety of additional functions and parameters depending on the make and model [13]. Building upon the capacity to self-monitor as an important component of behavior change theory, physical activity interventions designed with wearables have increased in number over the past few years [12,14,15]. Typically, these patient-oriented interventions include a wearable device with some type of human support delivered by a combination of telephonic, SMS text messaging, and in-person sessions that were either one-on-one or group-based [12,16-19].

A total of 2 recent meta-analyses of physical activity interventions with wearables have reported small effect sizes overall, with some increase in daily steps. Lynch et al [15] identified 21 studies that met the initial criteria; however, heterogeneity was quite extensive across the studies. The results of a subset of 9 studies that included a comparison group suggested that wearables contributed to an approximate increase of 500 steps/day. A second meta-analysis conducted by Franssen et al [20] focused exclusively on wearable activity tracker-based interventions in patients with a range of chronic cardiometabolic diseases. The results of this analysis suggested an average increase of 2100 steps/day following the interventions [20]. Neither analysis explored the impact of participants’ demographic characteristics on the response to the interventions. In fact, with few exceptions, studies evaluating wearables have not described the racial and ethnic backgrounds of the participants [21]. Those that do report this information typically have a small representation from these groups [22]. This is problematic considering that racial and ethnic minorities and those with lower socioeconomic status likely have the most to gain from increased engagement in physical activity.

### Objectives

The aim of this study is to pilot-test an 8-week text-based walking program with physically inactive, ethnic minority patients within a primary care setting. The primary outcomes included feasibility and acceptability. A mixed methods research approach was used, which included a summative evaluation that offered qualitative information on the patients’ experiences. The number of steps measured by Fitbit was analyzed as an estimate of the change in physical activity.

## Methods

### Participant Recruitment

Patients were recruited as a convenience sample within an internal medicine outpatient clinic that was part of an urban academic medical center serving a predominantly ethnic minority patient population. The research staff members were present in person. Recruitment relied on physicians and clinic staff who were aware of the study activities and referred patients presenting for a regularly scheduled appointment at their own discretion. However, they were asked to refer patients who were English-speaking, overweight, and healthy enough to increase their walking activity. As such, the sample was one of convenience. Once referred, the research staff screened patients for eligibility as follows: (1) self-reported that they engaged in <150 minutes of moderate to intense physical activity a week [23], (2) aged 21-65 years, (3) comfortable communicating in English, (4) not currently or planning to be pregnant, (5) had physician clearance to walk, (6) owned a smartphone with unlimited text messages, (7) sent text messages, (8) had no serious mental health issues, and (9) BMI>25 kg/m^2^. Only 1 participant per household was allowed to be recruited. Interested and eligible participants provided written informed consent. The consent informed patients that if an emergency arose, they should seek medical care directly and not rely on SMS text messaging the health coach (HC). All participants received the intervention. The enrollment goal was 30 participants.

### Fitbit and SMS Text Messaging Platform Setup

After completing an interviewer-administered demographic survey, participants received a Fitbit Charge 2 or Charge 3 and training on the device. The research staff downloaded the Fitbit app onto participants’ smartphones using a study-designated Gmail account. The Fitbit device was synchronized to the Fitbit app. Patients’ mobile phones and study identifiers were entered into Mytapp, a 2-way SMS text messaging platform used to communicate and set goals with patients. Mytapp is a custom-designed research platform that uses Fitbit’s web application programming interface to securely obtain physical activity data on participants (heart rate, steps, and battery status). Mytapp only provides a simple interface to visualize Fitbit data in graphical form at the time of request. No Fitbit data are ever stored on the Mytapp server. When HCs use Mytapp, they are able to view the most recent Fitbit data available on demand (based on the last time the user synchronized their Fitbit device).

All communication over the 8-week intervention period was transmitted through text messages sent on Mytapp. Text messages sent by the HCs were in free form. Participants were informed that replying with *STOP*, *QUIT*, *or END* would automatically block Mytapp from sending further messages without requiring contact from the research team. Participants who wished to withdraw from the study needed to explicitly indicate so in text or contact a research member.

### Health Coaches

Participants were assigned to 1 of the 3 HCs who were research team members unaffiliated with the clinic. The lead HC was an experienced health psychologist (LS), who formally trained two other coaches on all study activities, including integration into the primary care clinic, patient recruitment and technology setup, Mytapp platform use, and the goal-setting process that supported participants setting their own goals. Coaches were responsible for tracking their panel of patients and monitoring the text messages daily.

### Goal-Setting Activities

Goal-setting followed the specific, measurable, attainable, realistic, and time-bound (SMART) goal framework. Participants were instructed that they would exchange a series of text messages with the HC to set a new step goal using the SMART goal framework each week. This was explained to patients in person at the time of recruitment and used in the process of setting the goal for week 1. They also received a handout that explained the SMART goal process in a low literacy language. To confirm that the messaging platform was successfully linked to their mobile phone, the staff set up the first goal through a text message with the patient. Finally, patients were told the name of their HC before leaving the clinic. Assignment to 1 of the 3 HCs was nonrandom. Within 1 hour of the patient leaving the clinic, the assigned HC was notified of a new patient and their first goal. Within 24 hours, the HC sent a text message to the patient with an introduction and welcome while confirming the set goal.

### Ethics Approval

The study was approved by the institutional review board at the University of Illinois at Chicago (institutional review board protocol number: 2016-0772).

### Intervention

All participants received the intervention in this pilot study. The walking program used 3 behavior change strategies found to be effective in increasing physical activity, including goal-setting, biofeedback, and prompts or cues to action [24].

Increased daily steps were theorized to be driven by individuals setting their personal SMART goal, which outlined explicit elements to increase success, such as time and location for walking, along with the number of steps. Participants set goals that they felt were realistic and achievable. HCs assisted the participants’ assessment of this by assessing their confidence in reaching the goal on a scale from 0 to 10. Goals rated <8 were revised to increase the confidence of the participants. Fitbit devices provided immediate real-time feedback on steps, with HCs supporting the feedback through SMS text messaging, which reinforced the participants’ goals as well as offering cues to action. In addition, the HC provided supportive accountability and social support for goal attainment.

For 8 weeks, the assigned HC sent text messages related to walking goals (eg, “Only 500 more steps to reach your goal for today!”). The participant set a new SMART goal with HC support each week via SMS text messaging. Goal-setting was expected to require several text message exchanges that could extend over a day or more. If the participant did not get a new goal set up within the 7-day week window, the subsequent week window began once the new goal was set. The HCs sent a minimum of 5 text messages each week. Additional text messages from the HC responded to patient-initiated text messages, troubleshot synchronizing failures with the Fitbit, or reminded the participants to charge their Fitbit batteries.

### Intervention Fidelity and Safety Monitoring

To ensure HC fidelity to using the SMART goal framework with participants, HCs met weekly to review all text message exchanges. A psychologist with previous experience in the goal-setting framework reviewed the text messages with the team. Feedback and discussion focused on ensuring that all aspects of the SMART framework were addressed. In addition, standardized approaches to addressing goal-setting text messages were reviewed, with a focus on participants who had a delay in responding. Engagement in the program was defined as setting at least six goals over 8 weeks. Fitbit use was defined as participants having steps recorded within a 24-hour period in the Fitbit app. At enrollment, participants were instructed not to rely on the HC or SMS text messaging for medical emergencies. The HCs were instructed to report any concerning text message to the head psychologist immediately.

### Outcome Measures

The feasibility was assessed using several parameters. A fundamental indicator was the number of patients referred to the program, the number of patients who completed eligibility screening, and the number of patients who enrolled in the study. Engagement was operationalized as the number of weeks out of 8 weeks when a text message goal was set. The goal for engagement was 7 out of 8 weeks. Although no specific goal was set, the number of text messages sent and received by each participant and HC served as an additional descriptor of engagement. All text messages were stored in Mytapp. Retention was defined as the number of participants who set a goal in the last week of the program. Feasible retention was set at 80% for this pilot study. The feasibility of using the Fitbit trackers and Mytapp text messaging platform was assessed by maintaining a log of issues that arose during all phases of the program.

The acceptability of the program was assessed through a summative evaluation using qualitative methods and a 9-item standardized semistructured interview following the 8-week program. The summative evaluation addressed participants’ experiences with the program, including their motivation to participate, the goal-setting process, the frequency of SMS text messaging, Fitbit devices, and HCs. Research assistants conducted interviews in person or by telephone, depending on the patient’s availability. The interviews were audio recorded and transcribed using written notes. After the interview, the participants received a monetary compensation of US $50 for their time and retained their Fitbit device after the study.

Preliminary exploratory outcomes included average daily steps and duration of physical activity categorized as sedentary, lightly active, or moderately to vigorously active times (in minutes) as recorded in the Fitbit app. The pilot study was not powered to detect differences in the outcomes.

### Analysis

Descriptive statistics were calculated for demographic characteristics and the average number of text messages sent by the participants. Exploratory statistical analysis of the Fitbit data was performed using SAS (version 9.4; SAS Institute). After ensuring that the Fitbit step data were normally distributed, a paired *t* test (2-tailed) was conducted on the average weekly steps for weeks 1 and 8. Daily steps over time were explored using a linear mixed model accounting for repeated measures, similar to the methods outlined in the study by Polgreen et al [25]. The duration of the study began at enrollment and continued until the end of the study period (ie, after 8 weeks) or study dropout. Participants who had a significant positive or negative association between steps and days were categorized as having an increase or decrease in daily steps, respectively. Those who had no significant association between steps and days were categorized as having no change in daily steps. The patient’s average weekly steps were plotted across 8 weeks. The average of these values for all patients within each category (ie, whether they had an increase, decrease, or no change in their daily steps) was also plotted across 8 weeks. Changes in the average length of sedentary, lightly active, and moderately to vigorously active time in minutes from week 1 to week 8 were evaluated using a paired *t* test, with 95% CIs also reported. Analyses of whether patient demographics and patient engagement were associated with being categorized as having increased daily steps were conducted using Fisher exact test because of small cell sizes. All statistical tests were evaluated at a significance level of 5%. Missing data were handled in a conservative manner by assigning 0 steps walked that day for days where steps were missing. This conservative assumption was based on the patient population self-reporting low physical activity levels.

Qualitative data from summative evaluation were coded and managed using QDA Miner Lite (version 5; Provalis Research) [26]. A total of 2 coders and a qualitative expert read all the interviews before the meeting to develop a coding scheme using thematic analysis. A total of 5 interviews were independently coded by the 2 coders, followed by reviewing and refining the codes by the qualitative expert. The coders independently applied the coding to the remaining interviews. Subsequently, the coders and the expert met to review all codes, resolve any discrepancies, and identify relevant themes.

## Results

### Baseline Characteristics

Participants were recruited from May 2019 to August 2019. Participant demographics and baseline characteristics are shown in Table 1.

Of the 30 participants, 23 (77%) participants were women and were aged on average 47 years (SD 9.6 years). A total of 80% (24/30) of participants were African American, and 17% (5/30) were Hispanic or Latino. A total of 20% (6/30) of participants reported a yearly household income <US $20,000, and 37% (11/30) of participants reported high school as their highest level of education. At baseline, the average BMI was 39.3 kg/m^2^ (SD 9.3 kg/m^2^) and 77% (23/30) of the participants were considered obese (BMI≥30 kg/m^2^) [27]. A total of 30% (9/30) of participants self-rated their health as either *fair* or *poor*.

**Table 1:** Demographic and baseline characteristics (N=30).

Characteristics			Value
**Baseline BMI, mean (SD)**			39.3 (9.3)
	Overweight (25.0 kg/m^2^>BMI<30 kg/m^2^), n (%)	5 (18)	
	Obesity class 1 (30 kg/m^2^>BMI<35 kg/m^2^), n (%)	7 (25)	
	Obesity class 2 (35 kg/m^2^>BMI<40 kg/m^2^), n (%)	2 (7)	
	Obesity class 3 (BMI≥40 kg/m^2^), n (%)	14 (50)	
Age (years) at enrollment, mean (SD)			47.1 (9.6)
Gender (female), n (%)			23 (77)
**Race, n (%)**			
	African American or Black	24 (80)	
	Hispanic or Latino	5 (17)	
	White and non-Hispanic	1 (3)	
**Health insurance, n (%)**			
	Health Maintenance Organization or Preferred Provider Organization	19 (63)	
	Medicare	2 (7)	
	Public aid or Medicaid	9 (30)	
**Employment, n (%)**			
	Worked full-time for pay	18 (60)	
	Worked part-time for pay	5 (17)	
	Disabled and unable to work	3 (10)	
	Out of work or unemployed	3 (10)	
	Retired	1 (3)	
**Highest level of education, n (%)**			
	High school diploma or General Educational Development	12 (40)	
	2-year certificate or associate degree	9 (30)	
	College graduate	9 (30)	
**How would you describe your health? n (%)**			
	Excellent	1 (3)	
	Very good	7 (23)	
	Good	13 (43)	
	Fair	8 (27)	
	Poor	1 (3)	
**Marital status, n (%)**			
	Married or living with partner	9 (30)	
	Divorced or widowed	3 (10)	
	Single, never married	18 (60)	
**Yearly household income (US $), n (%)**			
	<19,999	6 (20)	
	20,000-49,999	9 (30)	
	50,000-69,999	8 (27)	
	≥70,000	6 (20)	
	Refused to answer	1 (3)	

### Feasibility

#### Recruitment and Retention

A total of 33 patients were referred by primary care physicians to the research staff. All 33 patients were screened for eligibility, of which 1 patient was ineligible because of the age criteria and 2 patients were not interested after screening. In total, 91% (30/33) of patients were enrolled in the study. The study’s retention rate was 93% (28/30), exceeding the minimum 80% threshold. One individual withdrew from the study after week 6. Another individual stopped responding to the text messages from the HC starting week 4 and was withdrawn per established protocol. One individual lost their Fitbit device after week 4 but participated in the summative evaluation. Two of the first patients recruited received a Fitbit Charge 3, which the research staff were unable to synchronize to the participants’ mobile phone app or with the Mytapp app. As a result, HCs were not able to monitor the steps for these 2 patients. However, they set up weekly step goals with the HCs through SMS text messaging and self-monitored their daily steps on their Fitbit devices for the entire study. Charge 2 models were used on all subsequent participants without initial synchronizing problems.

#### Engagement Through SMS Text Messaging

As the 2 patients who withdrew from the study contributed to <2 weeks of goal-setting text messages, they were excluded from the SMS text messaging analysis (decided a priori).

The average number of weekly text messages sent by 28 patients and their HCs each week is shown in Table 2.

**Table 2:** Average weekly text messages sent by patients and coaches across 8 weeks (N=28)^a^.

Week	Patients, mean (SD)	Health coaches, mean (SD)
1	11 (5.9)	14.9 (7.4)
2	10.9 (7.7)	14.2 (8.5)
3	7.8 (5.4)	11.1 (5.5)
4	7.1 (3.9)	11.3 (5.3)
5	5.6 (3.1)	8.8 (4.7)
6	6.9 (5)	9.5 (4.7)
7	6.6 (4.4)	9.2 (5.2)
8	6.7 (3.3)	9.2 (3.6)

^a^Two patients were excluded from this analysis because they contributed to <2 weeks of goal-setting text messages.

The average number of text messages sent by HCs was significantly greater than the number sent by the participants (*P*=.02) as expected. HCs sent reminder and motivational text messages that did not require a response. Interestingly, the difference remained fairly stable across time with HCs sending, on average, 3 to 4 more text messages than the participants, despite the average decrease over time for both groups. There were 73% (22/30) of patients who set at least seven goals across the 8 weeks, with 47% (14/30) patients setting a goal every week. A total of 13% (4/30) of patients set 6 goals, 3% (1/30) of patients set 5 goals, and 10% (3/30) of patients set 3 goals or less.

### Acceptability

A total of 93% (28/30) of participants completed the exit interview, which lasted between 15 and 30 minutes. Owing to availability, telephone interviews were completed with 3 participants. Textbox 1 shows a summary of the 10 key themes that emerged when participants were asked to describe their experience with the pilot program and exemplary quotes.

**Themes and quotes from qualitative interviews of patient experiences with the pilot program.**

**Patient motivation**

- “I also wanted to walk more to help with my anxiety and depression.” [Female participant, aged 42 years]
- “I wanted to lose weight. I didn’t like how much I weighed. I wanted someone to keep me accountable.” [Female participant, aged 41 years]
- “[I wanted] to exercise more, keep moving; be a little bit healthier. The study helped me to achieve that.” [Male participant, aged 55 years]

**Convenient texting experience**

- “It was more convenient for me. I didn’t have to take time from work to set those goals and go all the way to go into a location to talk to a HC or have to have a long conversation to talk to a HC because it was like between the time I was going to work or getting ready to start work, so I was able to text back.” [Female participant, aged 48 years]

**Social support**

- “I felt like I was really connected to a person that was supporting me and encouraging me to continue to take my steps.” [Female participant, aged 48 years]

**Supportive accountability**

- “When you are only accountable to yourself, you can blow [the goal] off; but when you are accountable to someone else...I don’t want her (the HC) to see I am not doing anything” [Male participant, aged 55 years]
- “If I got so caught up with my everyday workload, and I checked my phone with the coach...it triggered me to touch bases with the HC...the texting and communication kept me kind of focused on the goals even though I had a lot of distractions.” [Female participant, aged 58 years]

**Technology support**

- “When my Fitbit wasn’t syncing she [the HC] would reach out” [Female participant, aged 34 years]

**Self-determined goals**

- “...the understanding [and] acceptance if the goal went down or went up or whatever, it was all up to me.” [Female participant, aged 42 years]
- “I knew you (the HC) was going to be texting me to see what my goal was, and I tried to think of it before, so I was ready, but it was hard sometimes.” [Female participant, aged 45 years]

**Achievable goals**

- “Everything was accomplishable and reasonable. It (the step goal) was realistic. You [the HC] always said make it something that you know you can succeed. You weren’t really giving me something high and big that I might struggle with.” [Female participant, aged 45 years]
- “No one was pressuring you to go higher or lower. I really enjoyed that.” [Female participant, aged 33 years]

**Feedback from Fitbit device**

- “I never paid attention to how many steps I took. So, with this [the Fitbit], it made me focus or pay attention.” [Female participant, aged 49 years]
- “It was cool to see how many steps I got and to try to get some more.” [Male participant, aged 44 years]

**Challenges**

- “The texting didn’t give a push. It was a little push because you are talking to someone, but it wasn’t like someone yelling at you to work out. Hands-on is always better. But the texting is fine; it’s just not hands-on.” [Male participant, aged 48 years]

**Habit formation**

- “Usually I wouldn’t do it [walking]. But now it’s a habit, and if I don’t see the texts, I have the Fitbit.” [Female participant, aged 45 years]

Nearly all participants expressed that they enrolled in the study with a desire to improve their health, increase their physical activity, or lose weight. Some were curious about their daily step count. Although most appreciated the ability to control the goal they set each week, 3 participants experienced the self-setting goals to be challenging and commented that they would have liked the HC to set the goals. Importantly, many participants commented that they felt their goal could be reached successfully. Setting realistic goals was 1 component within the SMART goal framework, thereby supporting the fidelity of the goal-setting process. The participants identified a few barriers to study involvement. Almost half indicated that their busy schedules hindered their ability to send back a text message to the HC immediately. As a result, lag periods between receiving text messages from the HCs and responding to them were common, ranging from hours to days. When the content is related to goal-setting, HCs typically sent another text message the following day. However, many text messages did not require responses; therefore, the delay was of little consequence. The participants also noted some problems related to the technology. One Fitbit band broke and was replaced quickly but required the participant to travel to the study site to pick it up. A few participants experienced synchronizing issues among the Fitbit, mobile app, and text messaging platform. Furthermore, 2 participants noted that while sending a text message to an HC was convenient, they preferred face-to-face or verbal communication, pushing them directly to work out harder. In addition, patient-reported barriers to attaining step goals included forgetting to put the Fitbit device on in the morning, not adhering to their goals on the weekends, and acute health-related problems. Finally, suggestions for improving the program included adding reminders to eat healthily, providing goal suggestions, and incorporating mutual competitiveness among participants.

In addition to the 10 themes, participants noted additional behavior changes related to focusing on their step goals. Examples include self-reported weight loss, perceived improvement in chronic health conditions, and overall increased health consciousness. Despite the fact that the program did not have any content related to other health behaviors, some mentioned that they had increased fruit and vegetable consumption or decreased late night eating. One participant reported that she started walking with her husband to gain benefits from increased activity.

### Safety

One safety issue required follow-up. A text message from 1 participant mentioned that they were unable to meet their step goals because of *pain from* falling. This was immediately reported to the lead HC who contacted the participant. The participant had fallen in their backyard completely unrelated to walking as part of the program. The pain was from twisting an ankle that the participant reported was minor. No further action was required, and the participant was working on step goals within 2 days.

### Exploratory Analysis of Fitbit Steps Data

Fitbit data from all 30 participants were included in the analyses. There was no significant change in the average weekly steps for week 1 compared with the average weekly steps for week 8 (mean difference 7.26, 95% CI −867.5 to 882.0). The linear mixed model showed that there were 17% (5/30) of participants who had a significant increase and 20% (6/30) of participants who had a significant decrease in their daily steps. The remaining 61% (17/30) of participants showed no change in their daily steps. There were no statistically significant changes in average weekly sedentary minutes (mean difference −17.6, 95% CI −67.8 to 32.6), average lightly active minutes (mean difference −3.37, 95% CI −28.8 to 22.1), and average vigorously active minutes (mean difference 6.79, 95% CI −3.4 to 17.0) from week 1 to week 8 among the cohort of participants. There was no association between baseline demographic characteristics and increase in their average daily steps. Similarly, there was no association between engagement (measured by the number of text messages) and increase in their average daily steps. The average weekly steps across the 8-week program for patients who increased and decreased their steps over time can be seen in Multimedia Appendices 1 and 2.

## Discussion

### Principal Findings

The feasibility and acceptability of a walking program designed around remote text-based goal-setting with an HC was supported among a sample of ethnic minority patients with chronic health problems, including overweight and obesity. The sample was diverse in terms of self-reported health status, including approximately one-third with fair or poor health status. Patients’ interest in the study was extremely high, with 91% (30/33) of those referred by their physician successfully enrolled in the study. Patients were able and willing to engage in SMS text messaging with the HC to set walking goals using the SMART framework over 8 weeks. Poststudy interviews suggested that participation was driven by motivation to improve their health both psychologically and physically. The salience of this motivation may have been heightened by the fact that patients were approached after an appointment with their primary care provider, which serves as a call for action to some patients [28,29].

Overall retention and text message engagement compared favorably with other studies. A recent meta-analysis of 35 studies testing wearables in people with cardiometabolic diseases reported an 87% mean retention (range 63%-100%) [12]. These studies ranged in length from 4 to 52 weeks, with 60% of the studies between 4 and 16 weeks. Despite continued engagement in this pilot study, the frequency of SMS text messaging decreased over time. However, given that the decrease in the frequency of SMS text messaging by the participants was consistently 3 to 4 SMS text messages less than the HCs frequency of SMS text messaging by the HCs, it is possible that the decrease was related to increased efficiency in applying the SMART goal framework. Future studies may explore this hypothesis by intentionally varying the frequency of HC text messages across time to assess whether this impacts the frequency of SMS text messaging by the participants.

Consistent with other reports, some Fitbit challenges had to be overcome [30]. Initially, participants were offered the choice of a Fitbit Charge 2 or Charge 3. However, research staff were unable to synchronize Charge 3 for 2 of the first 3 participants, and subsequent participants all received Charge 2 without setup issues. Occasional lags occurred when synchronizing the Fitbit with the app and text messaging platform. Some participants needed reminders to charge the device. This was facilitated by HCs monitoring the battery levels and sending text message alerts before the battery discharged. Having the HCs trained to address synchronizing or charging problems was important, as was training the participants when they received the Fitbit. A total of 2 participants had previous experience with Fitbit devices. However, the use of Fitbit devices and SMS text messaging was deemed feasible and acceptable in this population of socioeconomically diverse, ethnic minority samples.

This study is not alone in aiming to increase walking among people with chronic diseases by integrating a wearable (Fitbit) and health coaching [31]. However, this study is among the first to include a population that is disproportionately affected by chronic diseases and has unequal access to health technology. The sample comprised 97% ethnic minorities, of which 82% were clinically obese (BMI≥30 kg/m^2^) [27], and 30% had fair or poor self-reported health status. Despite the fact that existing evidence suggests that reaching moderate to vigorous activity levels are important, for many, this goal may simply be unrealistic. As noted by others, it is important to explore the impact of increased physical activity regardless of the intensity, particularly in individuals with physiological compromise because of chronic diseases [12,32,33].

This pilot study focused on refining a program by exploring its feasibility and acceptability. It was not designed or powered to explore the changes in the number of steps. Therefore, it is premature to draw conclusions regarding the efficacy of the program. However, HCs and text message content conveyed that some patients struggled to meet their goals because of acute health-related issues unrelated to the walking program. In such cases, HCs encouraged the participants to do only what they felt comfortable doing, regardless of their goal that week. In these cases, the HC checked in frequently to understand whether the health issue resolved and supported the participants’ return to walking goals when the participant felt better. Future work to develop this program would benefit from capturing patient health information at baseline and throughout the program to understand how health impacts participation in walking and guide adjustments in goals.

In understanding the experience of the participants, the most common theme that surfaced in the summative evaluation was social support and supportive accountability provided by the HC through the text messages. This suggests that the HC was a well-liked and important component of the program. In addition, almost two-thirds of the patients appreciated the convenience of SMS text messaging, which may have helped overcome certain barriers when it comes to engaging with an HC in real time (ie, appointments required for telephonic, in-person, or remote meetings). The asynchronous nature of SMS text messaging allowed both the patient and HC to freely respond on their own terms. This is highlighted where almost half of the patients indicated that their busy schedules hindered their ability to send back a text message to the HC immediately.

### Limitations

The generalizability of the study results is limited by several factors. The sample is not representative of the primary care populations. Recruitment was dependent on primary care physicians referring interested patients to the research staff. This process may have introduced selection bias. Nevertheless, as in previous research, our pilot study was able to successfully recruit by incorporating practitioners’ involvement [34]. In addition, the study used a within-person design and was not designed or powered to compare changes in the number of steps. Detailed medical histories were not obtained; therefore, the relationships among specific health conditions, goals, and steps cannot be explored. Over half (n=17) of the participants averaged 5000 steps per day in the first week, which may have been impacted by knowing that an HC would be monitoring their steps (ie, Hawthorne effect) or by having a novel Fitbit. Regardless, the range of steps was consistent with previous reports of step count among older patients or those with comorbidities or disabilities [9]. Finally, the continued development of the program must consider factors that may influence its dissemination and implementation in other settings. For example, the HCs in the study were trained research members. Not all primary care clinics have in-house HCs or clinical staff with the capacity to execute the key features of the program. Therefore, future work will need to consider factors, such as program effectiveness and adaptability as well as organizational characteristics that might influence its impact [35].

### Conclusions

The acceptability and feasibility of this remotely delivered walking intervention was supported among physically inactive underserved populations with chronic health conditions, such as obesity. Specifically, 91% (30/33) of the patients who were offered the study agreed to participate. Complete Fitbit and SMS text messaging data were collected from 93% (28/30) of the participants, with no adverse outcomes reported. Responses to the qualitative summative evaluation highlighted the convenience of remote delivery and social support experienced through the study. The results of the study support the inclusion of low-income and populations of color in the design of remote health interventions. Future research using a larger sample size and randomized control design is required to explore the efficacy of the approach to alter steps or perhaps, more importantly, to explore the impact on health outcomes and quality of life.

## Appendix

Multimedia Appendix 1 Average weekly steps for patients who increased their steps over time. The patient, noted with an asterisk, requested to withdraw during week 6.

Multimedia Appendix 2 Average weekly steps for patients who decreased their steps over time. Patient LKS08, noted with an asterisk, lost their Fitbit after week 4. Patient NAN14, noted with an asterisk, stopped responding to the text messages from their health coach starting week 4.

## Abbreviations

HC: health coach
SMART: specific, measurable, attainable, realistic, and time-bound

## References

[1] Boersma P, Black LI, Ward BW (2020). Prevalence of multiple chronic conditions among US adults, 2018. Prev Chronic Dis.

[2] Frayar C, Carroll M, Afful J (2020). Prevalence of overweight, obesity, and severe obesity among adults aged 20 and over: United States, 1960–1962 through 2017–2018. National Center for Health Statistics.

[3] Piercy KL, Troiano RP (2018). Physical activity guidelines for Americans from the US Department of Health and Human Services. Circ Cardiovasc Qual Outcomes.

[4] Booth FW, Roberts CK, Laye MJ (2012). Lack of exercise is a major cause of chronic diseases. Compr Physiol.

[5] (2020). Adult physical inactivity prevalence maps by race/ethnicity. Centers for Disease Control and Prevention.

[6] Warburton DE, Bredin SS (2017). Health benefits of physical activity: a systematic review of current systematic reviews. Curr Opin Cardiol.

[7] Stewart RA, Benatar J, Maddison R (2015). Living longer by sitting less and moving more. Curr Opin Cardiol.

[8] Stamatakis E, Gale J, Bauman A, Ekelund U, Hamer M, Ding D (2019). Sitting time, physical activity, and risk of mortality in adults. J Am Coll Cardiol.

[9] Tudor-Locke C, Craig CL, Aoyagi Y, Bell RC, Croteau KA, De Bourdeaudhuij I, Ewald B, Gardner AW, Hatano Y, Lutes LD, Matsudo SM, Ramirez-Marrero FA, Rogers LQ, Rowe DA, Schmidt MD, Tully MA, Blair SN (2011). How many steps/day are enough? For older adults and special populations. Int J Behav Nutr Phys Act.

[10] van der Wardt V, di Lorito C, Viniol A (2021). Promoting physical activity in primary care: a systematic review and meta-analysis. Br J Gen Pract.

[11] Brickwood K, Watson G, O'Brien J, Williams AD (2019). Consumer-based wearable activity trackers increase physical activity participation: systematic review and meta-analysis. JMIR Mhealth Uhealth.

[12] Kirk MA, Amiri M, Pirbaglou M, Ritvo P (2019). Wearable technology and physical activity behavior change in adults with chronic cardiometabolic disease: a systematic review and meta-analysis. Am J Health Promot.

[13] Henriksen A, Haugen MM, Woldaregay AZ, Muzny M, Hartvigsen G, Hopstock LA, Grimsgaard S (2018). Using fitness trackers and smartwatches to measure physical activity in research: analysis of consumer wrist-worn wearables. J Med Internet Res.

[14] Michie S, Abraham C, Whittington C, McAteer J, Gupta S (2009). Effective techniques in healthy eating and physical activity interventions: a meta-regression. Health Psychol.

[15] Lynch C, Bird S, Lythgo N, Selva-Raj I (2020). Changing the physical activity behavior of adults with fitness trackers: a systematic review and meta-analysis. Am J Health Promot.

[16] Li LC, Feehan LM, Xie H, Lu N, Shaw C, Gromala D, Aviña-Zubieta JA, Koehn C, Hoens AM, English K, Tam J, Therrien S, Townsend AF, Noonan G, Backman CL (2020). Efficacy of a physical activity counseling program with use of a wearable tracker in people with inflammatory arthritis: a randomized controlled trial. Arthritis Care Res (Hoboken).

[17] Barrett S, Begg S, O'Halloran P, Kingsley M (2020). A physical activity coaching intervention can improve and maintain physical activity and health-related outcomes in adult ambulatory hospital patients: the Healthy4U-2 randomised controlled trial. Int J Behav Nutr Phys Act.

[18] Hickey AM, Freedson PS (2016). Utility of consumer physical activity trackers as an intervention tool in cardiovascular disease prevention and treatment. Prog Cardiovasc Dis.

[19] Cadmus-Bertram LA, Marcus BH, Patterson RE, Parker BA, Morey BL (2015). Randomized trial of a Fitbit-based physical activity intervention for women. Am J Prev Med.

[20] Franssen WM, Franssen GH, Spaas J, Solmi F, Eijnde BO (2020). Can consumer wearable activity tracker-based interventions improve physical activity and cardiometabolic health in patients with chronic diseases? A systematic review and meta-analysis of randomised controlled trials. Int J Behav Nutr Phys Act.

[21] Nyenhuis SM, Balbim GM, Ma J, Marquez DX, Wilbur J, Sharp LK, Kitsiou S (2020). A walking intervention supplemented with mobile health technology in low-active urban African American women with asthma: proof-of-concept study. JMIR Form Res.

[22] Zytnick D, Kumar GS, Folta SC, Reid KF, Tybor D, Chomitz VR (2021). Wearable activity monitor use is associated with the aerobic physical activity guidelines and walking among older adults. Am J Health Promot.

[23] Piercy KL, Troiano RP, Ballard RM, Carlson SA, Fulton JE, Galuska DA, George SM, Olson RD (2018). The physical activity guidelines for Americans. J Am Med Assoc.

[24] Howlett N, Trivedi D, Troop NA, Chater AM (2018). Are physical activity interventions for healthy inactive adults effective in promoting behavior change and maintenance, and which behavior change techniques are effective? A systematic review and meta-analysis. Transl Behav Med.

[25] Polgreen LA, Anthony C, Carr L, Simmering JE, Evans NJ, Foster ED, Segre AM, Cremer JF, Polgreen PM (2018). The effect of automated text messaging and goal setting on pedometer adherence and physical activity in patients with diabetes: a randomized controlled trial. PLoS One.

[26] (2016). QDA Miner Lite. 2.0.7. Provalis Research.

[27] (2021). Defining adult overweight and obesity. Centers for Disease Control and Prevention.

[28] Martin A, Fitzsimons C, Jepson R, Saunders DH, van der Ploeg HP, Teixeira PJ, Gray CM, Mutrie N, Euro F (2015). Interventions with potential to reduce sedentary time in adults: systematic review and meta-analysis. Br J Sports Med.

[29] Epiphaniou E, Ogden J (2010). Evaluating the role of life events and sustaining conditions in weight loss maintenance. J Obes.

[30] Balbim GM, Marques IG, Marquez DX, Patel D, Sharp LK, Kitsiou S, Nyenhuis SM (2021). Using Fitbit as an mHealth intervention tool to promote physical activity: potential challenges and solutions. JMIR Mhealth Uhealth.

[31] Gell NM, Grover KW, Savard L, Dittus K (2020). Outcomes of a text message, Fitbit, and coaching intervention on physical activity maintenance among cancer survivors: a randomized control pilot trial. J Cancer Surviv.

[32] Mohr DC, Schueller SM, Montague E, Burns MN, Rashidi P (2014). The behavioral intervention technology model: an integrated conceptual and technological framework for eHealth and mHealth interventions. J Med Internet Res.

[33] McPhee JS, French DP, Jackson D, Nazroo J, Pendleton N, Degens H (2016). Physical activity in older age: perspectives for healthy ageing and frailty. Biogerontology.

[34] Ngune I, Jiwa M, Dadich A, Lotriet J, Sriram D (2012). Effective recruitment strategies in primary care research: a systematic review. Qual Prim Care.

[35] Huebschmann AG, Leavitt IM, Glasgow RE (2019). Making health research matter: a call to increase attention to external validity. Annu Rev Public Health.

