# Supplementary figures and images for: A Remote Health Coaching, Text-Based Walking Program in Ethnic Minority Primary Care Patients With Overweight and Obesity: Feasibility and Acceptability Pilot Study
Source: JMIR Form Res. 2022 Jan 19;6(1):e31989. doi: 10.2196/31989 (PMC8811699; doi:10.2196/31989)

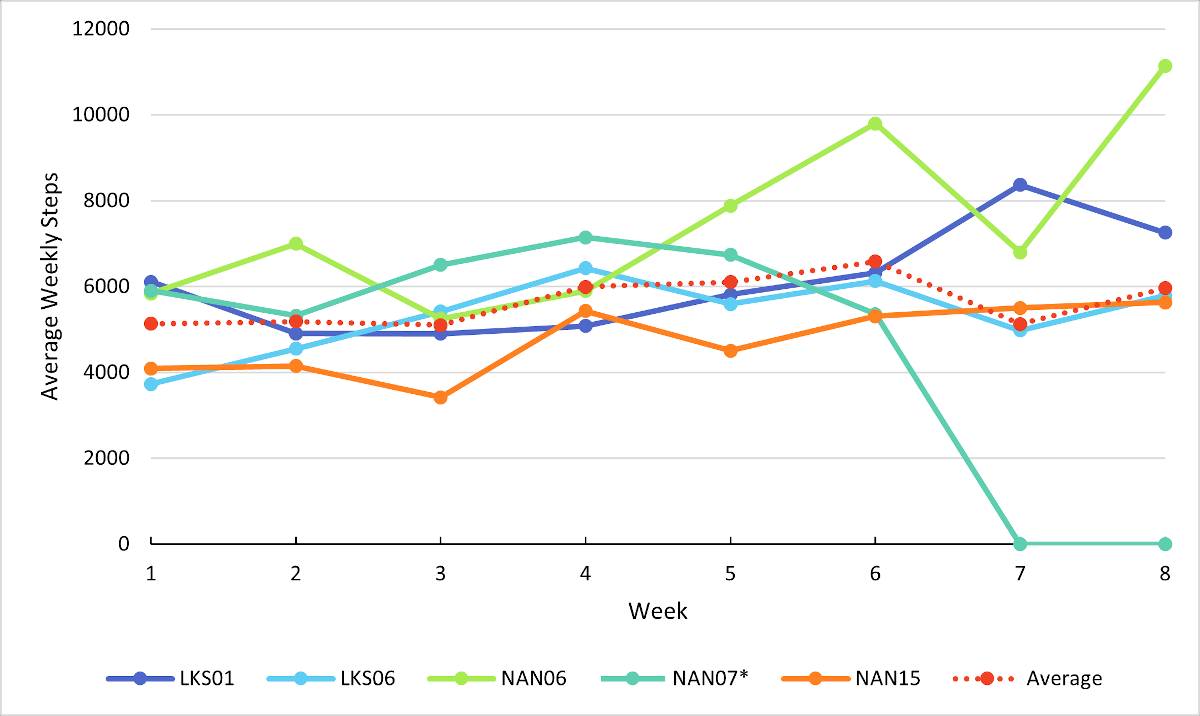

Supplement: Multimedia Appendix 1 [file formative_v6i1e31989_app1.png]

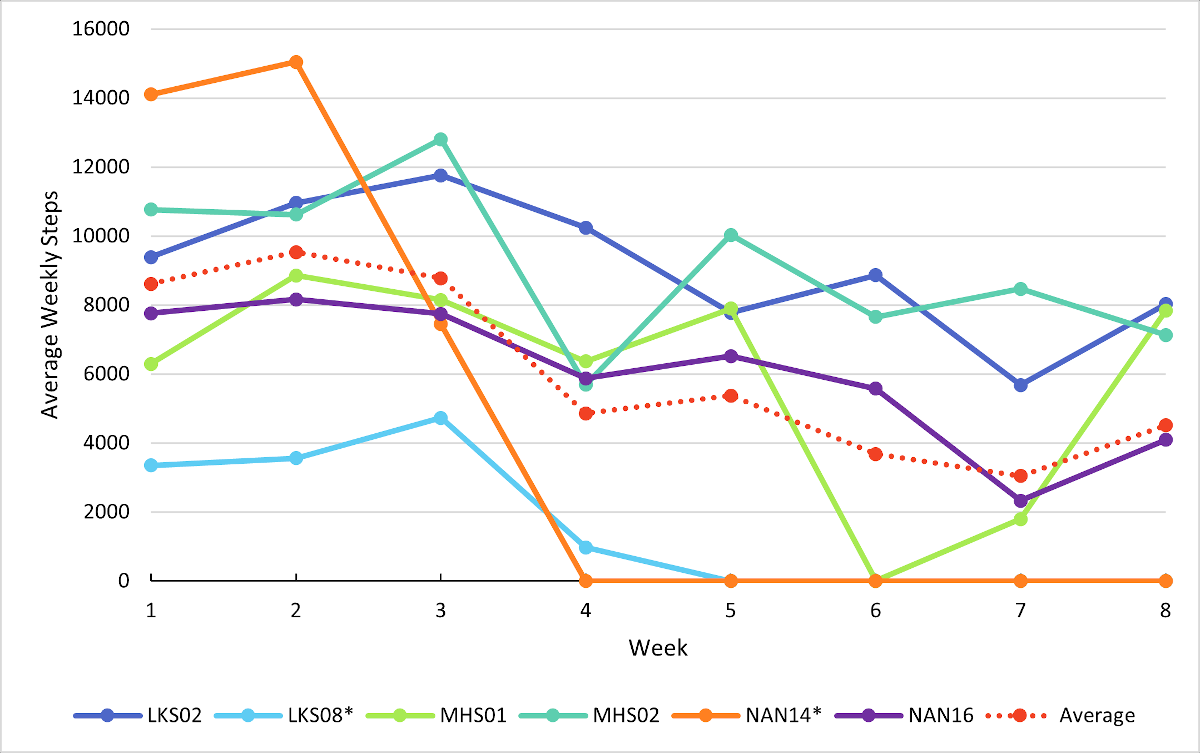

Supplement: Multimedia Appendix 2 [file formative_v6i1e31989_app2.png]
